# Extraction of Transcript Diversity from Scientific Literature

**DOI:** 10.1371/journal.pcbi.0010010

**Published:** 2005-06-24

**Authors:** Parantu K Shah, Lars J Jensen, Stéphanie Boué, Peer Bork

**Affiliations:** 1 Structural and Computational Biology Program, European Molecular Biology Laboratory, Heidelberg, Germany; 2 Max Delbrück Centre for Molecular Medicine, Berlin-Buch, Germany; University of California at San Diego, United States of America

## Abstract

Transcript diversity generated by alternative splicing and associated mechanisms contributes heavily to the functional complexity of biological systems. The numerous examples of the mechanisms and functional implications of these events are scattered throughout the scientific literature. Thus, it is crucial to have a tool that can automatically extract the relevant facts and collect them in a knowledge base that can aid the interpretation of data from high-throughput methods. We have developed and applied a composite text-mining method for extracting information on transcript diversity from the entire MEDLINE database in order to create a database of genes with alternative transcripts. It contains information on tissue specificity, number of isoforms, causative mechanisms, functional implications, and experimental methods used for detection. We have mined this resource to identify 959 instances of tissue-specific splicing. Our results in combination with those from EST-based methods suggest that alternative splicing is the preferred mechanism for generating transcript diversity in the nervous system. We provide new annotations for 1,860 genes with the potential for generating transcript diversity. We assign the MeSH term “alternative splicing” to 1,536 additional abstracts in the MEDLINE database and suggest new MeSH terms for other events. We have successfully extracted information about transcript diversity and semiautomatically generated a database, LSAT, that can provide a quantitative understanding of the mechanisms behind tissue-specific gene expression. LSAT (Literature Support for Alternative Transcripts) is publicly available at http://www.bork.embl.de/LSAT/.

## Synopsis

Given the functional complexity of higher eukaryotes, the relatively small number of genes in the human and other mammalian genomes came as a surprise to the scientific community. Later it was discovered that the majority of genes are subject to alternative splicing (“cutting and pasting”) or associated mechanisms that ultimately increase the diversity of transcripts that code for proteins. Studies exploring transcript diversity are currently dominated by high-throughput experiments and computational methods; however, the quality of such data should be assessed against a reliable reference set based on single-gene studies. Unfortunately, the latter type of information is scattered throughout the scientific literature. The authors have thus developed a computational approach for extracting information on alternative transcripts from MEDLINE abstracts and used it to create a database, LSAT. LSAT (Literature Support for Alternative Transcripts) provides information for more than 4,000 genes from about 14,000 abstracts. This database can provide a quantitative understanding of the mechanisms behind tissue-specific gene expression based on single-gene studies, which we show agrees well with EST-based studies (these studies involve tissue-specific splicing detected by the analysis of libraries of expressed sequence tags [ESTs]). These results indicate that mechanisms like alternative splicing, alternative promoters, and alternative polyadenylation work in concert to generate and regulate transcript diversity. More generally, information extraction of complex biological process seems feasible and can also complement large-scale data generation in other areas to assign functions to genes.

## Introduction

Although many model organisms have now been completely sequenced, we are still very far from understanding cellular function from genome sequence. One complicating factor is the expression of multiple alternative mRNA transcripts from a single gene using different mechanisms. Alternative promoters that are active in different tissues or at different developmental stages often regulate the expression of different mRNA isoforms, either directly through different transcription start sites or indirectly by promoter-directed exon inclusion in concert with alternative splicing (AS) [1]. Various AS mechanisms are known: alternative 5′ or 3′ sites can result in exons of different size, exons can be included or skipped, or an entire intron may be retained [2–5]. Alternative polyadenylation (AP), either alone or coupled with AS of 3′ terminal exons, may also generate transcript isoforms that are tissue- or developmental-stage-specific [6].

Generation of multiple alternative transcripts is important for the complexity and evolution of eukaryotic organisms [5,7–9]. In addition, their spatial and temporal expression patterns are believed to be one of the important factors behind the functional specificity of different tissues and organs. Moreover, defects in these processes are associated with various diseases [2]. Thus, developing an exhaustive catalog of alternative transcripts is a crucial task in order to fully understand the complexity of eukaryotes [7].

At present, high-throughput experiments and computational analyses dominate the mapping of the alternative transcript universe [10,11]. However, the quality and the biological meaning of these assignments should be assessed against a highly reliable benchmark set, which can be extracted from single-gene studies published in the scientific literature [3,12,13]. In addition, computational tools to explore the evolutionary conservation of mechanisms that generate transcript diversity (TD) are under development [14], which will also require a trustworthy set for rule learning.

Manual curation of experimentally determined biological events (physical interactions, AS, disease phenotypes, etc.) to generate trustworthy knowledge bases is slow compared to the rapid increase in the body of knowledge represented in the literature. Natural language processing tools thus play an increasingly important role in transferring information from free-form biomedical text to structured databases (see reviews [15–18]). This task can be split in to two steps: (1) a subset of documents describing events or scenarios of interest is identified (information retrieval [IR]), and (2) facts are extracted from these documents and deposited into structured fields (information extraction [IE]).

IR can be performed at the level of full articles, pertinent paragraphs, or sentences. As current IE methods operate at the sentence level, it may be appropriate to perform IR at the same level. Support vector machines have become the method of choice for IR tasks because of their ability to learn patterns and generalize well while handling large sets of input features, a common attribute of the text data [19–21]. Most IE systems use rules written by the domain experts to extract facts about events or scenarios of interest. The performance of most rule-based systems suffers because of the fact that any event or scenario can be written in one of many syntactically correct ways. Thus, an extraction system based only on syntactic patterns would require an exhaustive collection of rules in order to cover all possible patterns. The problem posed by multiple syntactic patterns can be solved by merging multiple syntactic patterns to a single semantic pattern by predicate–argument structures [22–24]. Predicate–argument structures and support vector machines (SVMs) are becoming prevalent in natural language processing and are widely believed to achieve good recall and precision; they were tested here for their applicability to the biomedical literature.

Here we present the benchmark and the results of a new extraction procedure that combines an SVM classifier with rule-based extraction of semantic patterns. The extracted knowledge about TD was stored in a database and subsequently used to quantify the amount of TD in different tissues. We discuss applications of our work for the assignment of MeSH terms (from the National Library of Medicine's Medical Subject Headings thesaurus), providing functional annotations to genes and to the transcript variants generated by computational methods.

## Results/Discussion

### Overall Strategy and Generation of the Database

**Figure 1:**
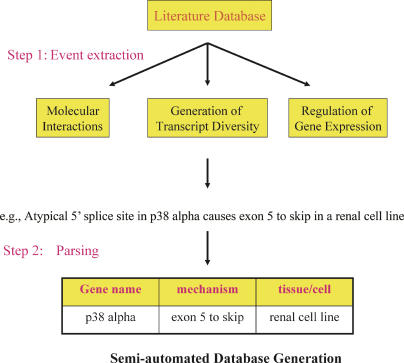
Creating Specialized Databases for Events of Interest A database of physiologically occurring AS events can be generated in two steps. Each step may involve machine learning or rule-based methods. The first step involves the identification of sentences from scientific text. These sentences can be parsed in a second step to extract frequently occurring semantic patterns.

To extract information about TD and associated spatiotemporal information scattered throughout MEDLINE, we devised a two-step procedure (Figure 1). In the first step, sentences containing TD information were identified within the papers' abstracts. To do so, and in order to overcome the problem of syntactic patterns, we tan SVM classifier for the sentence classification task by inductive machine learning [25] on an annotated corpus [19–21]. We then processed the entire MEDLINE database and identified sentences describing TD within those abstracts. In the second step, sentences were parsed and the word phrases were assigned different meaningful (semantic) categories (see Materials and Methods).

Finally, we mapped each abstract with information about alternative transcripts (retrieved by the SVM classifier) to entries in Swiss-Prot [26], RefSeq [27], GenBank [28], and Ensembl [29] databases, when possible. This not only provided the sequence information at genome, transcript, and protein level for the genes described in abstracts but also allowed us to access structural and functional information about these genes stored in various sequence databases. All this information obtained for each MEDLINE entry constitutes an entry in LSAT (Figure S1).

We identified eight different semantic categories describing biologically relevant data in the sentences describing TD, among which are event mechanism, species, tissue specificity, and experimental methods (Table 1; see Materials and Methods). In total we extracted 9,503 instances of event mechanisms from as many abstracts (Table S1) and 5,028 instances of tissues (Table S2) with associated gene names. Overall, the database contains 3,063, 874, and 207 nonredundant instances of AS, differential promoter usage (DP), and AP associated with genes and tissues extracted by entity taggers.

**Table 1:**
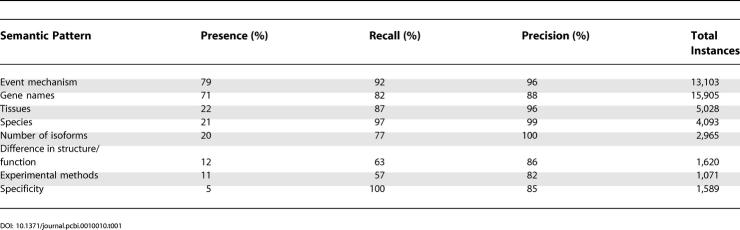
Extraction of Semantic Patterns

### Performance of the SVM Classifier for Sentence Retrieval

Our SVM classifier retrieved 31,123 putative TD-containing sentences from the MEDLINE database (12,948,515 abstracts). After false positives were removed by manual curation, 20,549 TD-containing sentences in 13,892 abstracts were left, corresponding to a precision of 66%. Details on the training set and SVM training procedure are described in Materials and Methods and Protocol S1.

We determined the recall of the classifier using manually curated AS annotations from MEDLINE and Swiss-Prot for annotations on human, mouse, rat, and *Drosophila*. All entries from MEDLINE 2004 annotated with the MeSH term “alternative splicing” and describing natural transcript generation (see Materials and Methods) were compared with our results. For each of these four species, we also analyzed our results on MEDLINE entries referred to in Swiss-Prot entries annotated with the keyword “alternative splicing” [26]. The average sensitivity of the classifier was 61% (Table 2; see Materials and Methods). The SVM classifiers thus achieve good recall and precision and can be used for extracting biological events.

**Table 2:**
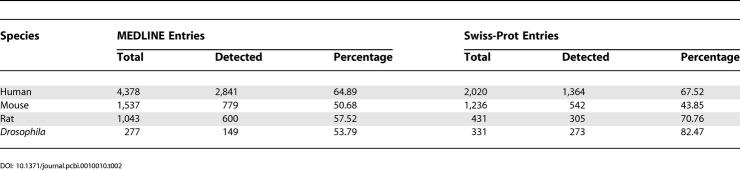
Recall of the SVM Classifier

### Performance of the IE Step

From the sentences retrieved by the SVM classifier, we extracted instances of eight semantic categories (see Materials and Methods) and evaluated the precision and recall by manually inspecting 300 randomly selected sentences for each category (see Table 1). Both precision and recall are highly satisfactory; however, it should be noted that accuracy in finding tag boundaries was not considered. Also, the recall is good for all categories, but not all eight categories are equally represented in the sentences (see Table 1).

### Proposing New Annotations in Curated Databases

Annotators at the National Library of Medicine have manually assigned the MeSH term “alternative splicing” to 8,133 abstracts. During the IE step, we identified 1,536 additional abstracts that mention AS but lack the MeSH term “alternative splicing,” corresponding to a 19% increase in annotation. We also identified DP and AP in 874 and 219 abstracts, respectively, for which we propose the new MeSH terms “alternative promoters” and “alternative polyadenylation” (Tables S3–S6).

We also quantified the number of Ensembl genes for which we can propose new annotations for AS (see Materials and Methods). The annotation increase observed was 20%, 52%, and 105% for human, mouse, and rat genomes, respectively (Figure S2). These tentative assignments can supplement the work of curators, and the numbers are likely to reflect the current extent of manual curation for these different genomes. The annotation increase for the human genes was relatively little compared to that for the rat genes because a total of 3,438 genes are already annotated in Swiss-Prot and RefSeq for AS in human, whereas only 342 genes are annotated for AS in rat. Even more annotations could be obtained by manually curating extracted events that could not be automatically mapped to a sequence database entry; we have manually mapped 190 genes exhibiting tissue-specific splicing. The observed increase in the annotation emphasizes the need for automated methods to speed up the process of database curation.

### Quantification of the Different Mechanisms That Lead to TD

The majority of vertebrate multi-exon genes undergo AS [10]. Moreover, different promoters may control the transcription of different mRNA isoforms, which may result in directed 5′ exon inclusion/exclusion, and AP signals can control the tissue specificity of alternative 3′ exons. While examples of synergy between these mechanisms are known, the extent of it is currently being explored. We found DP co-mentioned with AS in 14% of abstracts describing genes with differential promoters. A total of 19% of the abstracts providing information about alternative first exon usage also mentioned usage of different promoters. A total of 17% abstracts describing AP also mentioned AS.

**Figure 2:**
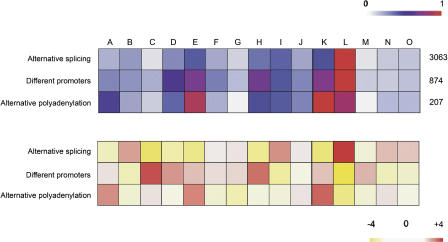
Preference for the Utilization of TD-Generating Mechanisms across Anatomical Systems Nonredundant instances of AS, DP, and AP are plotted against anatomical systems in which expression was found. The color of each square in the top panel signifies the ratio of the number of events detected for the system to the highest number of events within the row. Total number of nonredundant instances for each mechanism is on the left. The bottom panel shows the negative logarithm of *p*-values (see Materials and Methods for details). The anatomical systems are as follows: A, cardio vascular system; B, cells; C, connective tissues; D, digestive system; E, fetal/embryonic structures; F, endocrine system; G, exocrine glands; H, genitalia; I, immune system; J, integumentary system; K, musculoskeletal system; L, nervous system; M, respiratory system; N, sense regions; O, urinal system.

The extent to which various mechanisms are utilized for increasing TD may vary across different anatomical systems. To study this, we mapped all vertebrate tissue information to anatomical systems using the MeSH anatomy terms and counted the number of nonredundant events extracted for each mechanism in each system (Figure 2, top panel). AS is utilized equally in most organs except in the nervous system, where AS is significantly overrepresented (Figure 2, bottom panel). Similarly, there is significant overrepresentation of DP in the connective tissues and to a lesser extent in the digestive system and in the genitalia.

The information about alternative promoter usage linked with specific gene names and tissues extracted in this study is the largest such collection available, to our knowledge. We expect that it would provide a reliable dataset for development of computational methods for predicting tissue-specific promoter usage.

### Tissue-Specific Differences in the Extent of AS

AS has been shown to play an important role in creating functional specialization of tissues and development stages [30,31], but only a small number of instances of tissue-specific splicing are listed in the current AS databases [32,33]. With a large collection of high-quality AS events in hand, tissue-specific differences in AS should become visible. We checked entries in our database containing the field “specificity.” We identified 959 events describing tissue specificity in AS. These represented 675 AS events for pairs of tissues and 284 events where only one tissue was reported. The results contained 400 nonredundant events for 183 human genes. We also mapped a further 190 genes (not included above) from various species to Swiss-Prot identifiers during the manual curation.

**Figure 3:**
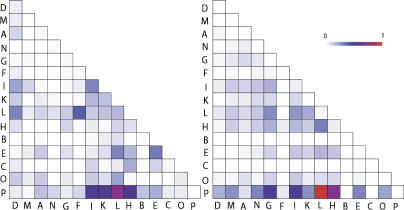
Tissue Specificity in AS The figure shows the body system distribution of differential/specific splicing. The instances were obtained from literature mining (left panel) and analysis of EST data ([33]; right panel). Each square is colored according to the ratio between the corresponding count and the highest count within the panel. Letter codes for anatomical systems are as in Figure 2. P represents a unique transcript.

To study the extent of tissue-specific AS, we mapped tissues and organs to respective systems as described in the previous section and plotted the results (Figure 3, left panel). The nervous system, genitalia, and immune, digestive, and musculoskeletal systems showed extensive tissue specificity in inter- and intra-systemic AS. These systems also showed expression of unique AS transcripts, with the nervous system showing the highest number of unique transcripts. These tissue-specific patterns of expression extracted from the literature strongly overlap with the 667 tissue-specific AS events derived from analysis of the EST data [33] for 454 human genes across 46 tissues (Figure 3, right panel).

The knowledge extracted from the literature confirms EST-based studies [31,33] and earlier experimental studies [34] that showed AS as the preferred mechanism for generating TD across the nervous system. EST-based studies [31] have also suggested that genes in liver (digestive system) and testis (genitalia) show distinct patterns of splicing with alternative exons. Our results indicate that these transcripts may show these different patterns of splicing in combination with different promoter regions. This conclusion seems plausible since AS of first exons is influenced by alternative promoter regions in at least 19% of cases (see above; [35]), and it should be explored further.

### Assigning Function to the Transcripts Generated by Computational Analysis

**Figure 4:**
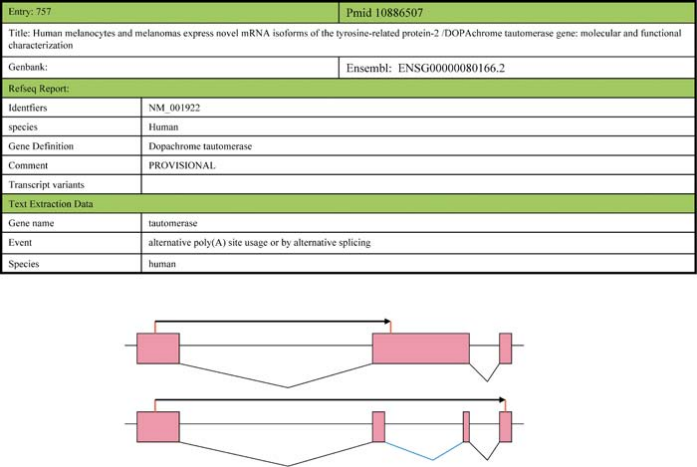
Assignment of Function Using Database Knowledge This figure shows a database entry that derives very little functional annotation from sequence databases. Text extraction rules were successful in identifying gene name, tissue, and event mechanism for the *Dopachrome tautomerase* gene. Multiple transcripts of the gene using SPLICE-POA [37] were produced by utilizing alternative 3′ splice sites and polyadenylation signals as speculated in the research article (bottom panel). Pink rectangles denote the exons, black lines describe constitutive splice sites, and blue lines show alternative splice sites. Black arrows show the different proteins generated via AS.

Sometimes experimental biologists speculate about the mechanism responsible for the multiple transcripts observed with a limited number of experiments but the corresponding transcripts are not deposited in GenBank. For example, work by Pisarra et al. [36] on human *Dopachrome tautomerase* describes two transcripts in melanocytes and melanomas with a “different carboxyl-terminus” generated, concluding that “dopachrome tautomerase can yield different isoforms by alternative poly(A) site usage or by alternative splicing” (Figure 4).

On the other hand, various methods, including those based on aligning EST and other sequence data to genomic regions, are currently in use for detecting AS on a large scale. The function of the isoforms thus generated is largely unknown [37], and these transcripts are poorly annotated in sequence databases.

Using the heaviest bundling algorithm [37] with genomic sequence data from Ensembl [38], and transcript data from UniGene [39] clusters for the gene, we were able to generate two transcript isoforms for *Dopachrome tautomerase* (Figure 4, bottom) resembling those described by Pisarra et al. [36] and were able to detect an AS event in the 3′ region. Hence, the use of large-scale methods may provide detailed information about underlying events, and text mining would provide functional annotations to the transcript isoforms observed.

### Conclusions

We successfully extracted information about the genes that express multiple transcripts and associated spatiotemporal information using state-of-the-art methods in natural language processing and utilized it for function annotations. The information extracted by far exceeds current manual curation efforts and generates reliable results. Our results indicate that mechanisms like AS, DP, and AP work in concert for the generation and regulation of TD. They also suggest that the nervous system preferentially relies on AS over other mechanisms to express the largest set of tissue-specific transcripts. In contrast, genitalia and the digestive system more frequently make use of alternative promoter regions. The knowledge stored in the database about synergy and preference for TD-generating mechanisms across tissues will be integrated to high-throughput data in the future. More generally, IE of complex biological processes seems feasible and can also complement large-scale data generation in other areas to assign function.

## Materials and Methods

### 

#### Training corpus and SVM learning.

A set of 4,240 sentences describing physiological TD and 13,520 negative sentences were selected as a training corpus from article titles and abstracts. Sentences describing mutations, clinical studies involving patients, nucleotide transversions, and splicing mechanisms were considered negative sentences. Sentences describing natural gene expression, gene paralogs, and aberrant transcripts showed word usage similar to those describing TD, making the classification task more challenging. Description of the learning corpus can be found in Protocol S1 and Figure S3.

The text in all the abstracts was split into sentences using the Oak system (S. Sekine, unpublished data; http://nlp.cs.nyu.edu/oak/). All the sentences were tagged with Tree-tagger [40] to give words their part-of-speech tags. Sentences were broken down into constituent words and stemmed to act as features to learn from. Stop words and words with certain part-of-speech tags were removed from the primary features. To add domain knowledge and enrich the features to learn from, frequently occurring word bi-grams and tri-grams were also defined from unprocessed sentences. The feature file was large, containing 23,742 features.

The procedure of inductive learning (see Protocol S1) was applied for the sentence classification task, using the feature vectors described above as input. We compared the performance of naïve Bayes, expectation maximization, maximum entropy, variants of TF-IDF, K-nearest neighbors, and support vector machines for the task [21,41–43]. The SVM with a radial basis function kernel (gamma = 1.5 and *C* = 100) outperformed other methods and SVM classifiers with linear and sigmoid kernel functions (P. K. Shah and P. Bork, unpublished data).

The classifier was trained to extract only the natural TD from the written text, as contrasted by aberrant transcripts that are caused by genetic changes. For consistency, we removed the 2,767 of the 8,133 MEDLINE entries with the MeSH term “alternative splicing” that also had the MeSH term “mutation,” had no abstract text, or had erroneous assignment of the MeSH term “alternative splicing.”

#### Definitions of precision and recall.

Precision and recall are used in IR to measure the performance of methods and they are defined as below. 

Recall = TP/(TP + FN); Precision = TP/(TP + FP) (1)

Where, TP, TN, FP, and FN denote true-positive, true-negative, false-positive, and false-negative elements according to a classification criterion.

#### Parsing of the sentences using semantic patterns.

An event or a scenario is described in a sentence via the combination of a predicate (normally a verb) and its arguments [22–24,44]. While the same biological relation can be described in many syntactically different ways, only a limited number of semantic categories (e.g., gene name or tissue name) may accompany the predicates (see Protocol S1 for further discussion). Therefore, at this step we can apply rule-based methods without much loss of sensitivity.

We constructed semantic patterns similar to those described in the PASBio database of predicate–argument structure [22]. These patterns match informative parts of sentences, e.g., *“gene* lacks exon *n* in *tissue.”* The Stanford lexical parser was used for parsing the sentences [45,46]. Sentence trees were viewed using the TigerSearch tool for generating extraction rules for taking the semantic patterns from sentences [47]. (See Protocol S1 for examples of rules.)

The success in assigning gene, species, and event mechanisms to abstracts is as follows (Figure S3). A total of 46% of all abstracts were directly mapped to literature entries in sequence databases such as Swiss-Prot, RefSeq, and GenBank. A further 15% of all abstracts were assigned gene names using a gene tagger [48], with the species name extracted from the sentences and/or from the MeSH terms mapped with the synonym list. However, only 54% of all abstracts could be unambiguously assigned to a unique species (see Figure 2, category A in lower right histogram). The rest of the abstracts may have had gene and species information but they could not be assigned to a sequence database. Tissues were tagged using a dictionary made of tissue lists from the Swiss-Prot and RefSeq databases. They were assigned to the relevant anatomical system (top level MeSH anatomy terms) using the MeSH browser. We have submitted these entries for manual curation to EMBL-EBI's Alternative Exon Database [32].

#### Quantifying the gain in gene annotation.

To quantify the gain in gene annotation, first we mapped sequence information to the MEDLINE identifiers from the SVM classification using literature entries in Swiss-Prot, RefSeq, and GenBank. Second, we mapped sequence-containing entries for human, mouse, and rat genes present in our results and in those databases to Ensembl gene identifiers using the EnsMart system. Then we compared our annotations to those of Swiss-Prot and RefSeq to identify genes that were missed during the manual curation of AS. Special care was taken to avoid annotations that may have arisen because of a single literature entry mapping to multiple database entries. Hence, these annotations were highly significant.

#### Associating TD-generating mechanisms with organ systems.

The significance of the association of each TD-generating mechanism with each organ system was evaluated using the hypergeometric distribution. We corrected these *p*-values for multiple testing by calculating the false discovery rate using the Benjamini-Hochberg formula [49]. We found 14 significant associations (out of 45) at a 5% false discovery rate, three of which were also significant at a 1% false discovery rate.

## Supporting Information

Figure S1 An Example Database Entry (1.7 MB TIF).

Figure S2 Distribution of the Results of the IE Step (4.6 MB TIF).

Figure S3 Description of the Training Set (60 KB PDF).

Protocol S1 Supplementary Text (112 KB PDF).

Table S1 Genes and Associated TD-Generating Mechanism (423 KB TXT).

Table S2 Genes and Tissues (120 KB TXT).

Table S3 Abstracts Describing AS (445 KB XLS).

Table S4 Abstracts Describing Alternative Promoters (76 KB XLS).

Table S5 Abstracts Describing Alternative Initiation (20 KB XLS).

Table S6 Abstracts Describing AP (29 KB XLS).

## Abbreviations

AP: alternative polyadenylation
AS: alternative splicing
DP: differential promoter usage
IE: information extraction
IR: information retrieval
SVM: support vector machine
TD: transcript diversity
