# Supplementary figures and images for: Extraction of Transcript Diversity from Scientific Literature
Source: PLoS Comput Biol. 2005 Jun 24;1(1):e10. doi: 10.1371/journal.pcbi.0010010 (PMC1183516; doi:10.1371/journal.pcbi.0010010)

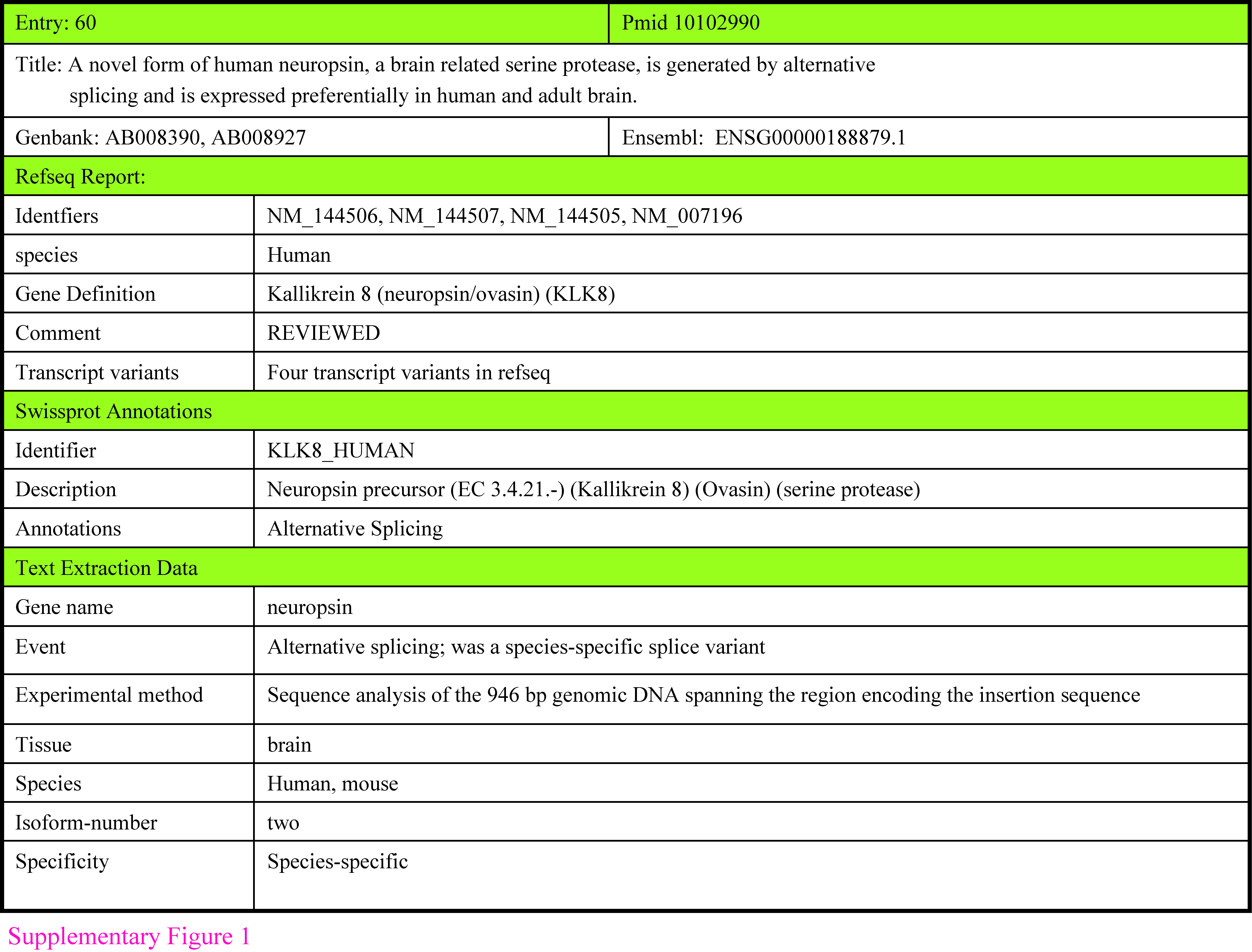

Supplement: Figure S1 — (1.7 MB TIF). [file pcbi.0010010.sg001.tif]

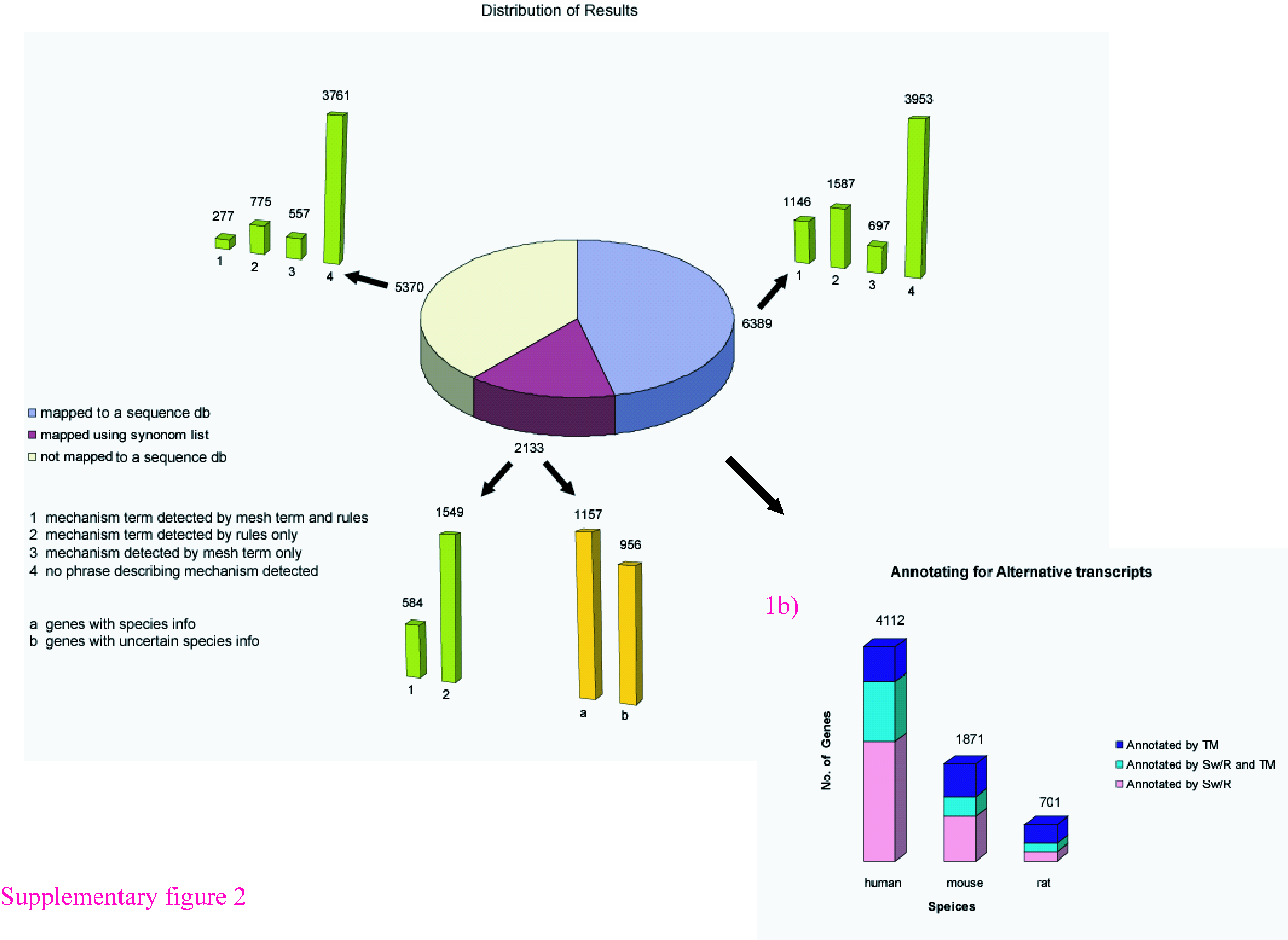

Supplement: Figure S2 — (4.6 MB TIF). [file pcbi.0010010.sg002.tif]
