# Supplementary material for: Extraction of Transcript Diversity from Scientific Literature
Source: PLoS Comput Biol. 2005 Jun 24;1(1):e10. doi: 10.1371/journal.pcbi.0010010 (PMC1183516; doi:10.1371/journal.pcbi.0010010)
Supplement: Figure S3 — (60 KB PDF). [file pcbi.0010010.sg003.pdf]

Supplementary figure 3

Figure 3a
Category 1

1. The [NAALADase/PSMA1]$_1$ gene is known to produce multiple [mRNA splice forms]$_2$ ([PSMA1a and PSMA1b]$_1$).
2. It had been assumed that the [human]$_3$ [brain and prostate]$_4$ express transcript variants of the enzyme generated by [differential promoter usage]$_2$.
3. One of these genes encodes [two]$_6$ different enzyme forms, alpha and beta, due to [the differential usage of first exons]$_2$.
4. The newly defined region contains [an intron that may be alternatively spliced and seven polyadenylation signal sequences]$_2$.
5. [EDNRDelta3]$_1$ generates the same amino acid sequence at the C terminus, but utilizes [the polyadenylation signal within the open reading frame]$_2$, resulting [in a shorter 3' UTR]$_5$.
6. The larger clone has 5' and 3' ends that are identical to the smaller clone but also has [an alternatively spliced 1.9-kilobase internal segment]$_2$.

Category 2

7. A [HPFK-M]$_1$ cDNA clone lacking [the sequences corresponding to exon IX]$_5$ was isolated from [human]$_3$ [fibroblast]$_4$ (IMR-90) library, suggesting that [HPFK-M]$_1$ transcript may be [alternatively spliced]$_2$.
8. Soluble [Fc gamma receptors]$_1$ are produced by [cleavage of the membrane receptors or by alternative splicing]$_2$.
9. [Northern hybridization analysis and RT-PCR]$_7$ suggests that the soluble and membrane bound forms of [human]$_3$ [AmP]$_1$ are products of [twodistinct genes or, through alternative splicing]$_3$, have different [C-terminal sequences]$_5$.

Category 3

10. In this study, we have identified [three]$_6$ [Skn-1]$_1$ isoforms, which encode [peptides with various N termini]$_5$.
11. These [two]$_6$ [hRPB3]$_1$ mRNA species differed in [3' UTR region length]$_5$, the longer transcript containing the AU-rich sequence motif that mediates mRNA degradation.
12. If this question is correct, the observed differences in [amino acid sequences]$_5$ could be explained by the existence of different mRNAs for gamma and gamma' chains.

13. [Northern blot analysis]$_7$ detected 2.4 kb and 3.2 kb mRNA transcripts in all tissues examined.
14. [Gene expression analysis using cph genomic fragments from normal and neoplastic cells]$_7$ identifies a number of transcripts including a major mRNA of 2.5 kb as well as several smaller transcripts.
15. There were [tissue-specific]$_8$ differences in the size of mRNA transcripts in [human]$_3$ [brain]$_4$ tissues as well.
16. All [six]$_6$ mRNAs were present in the samples analyzed.

figure 3b

17. A G to T mutation in exon 6 results in an in-frame termination codon in eight Hispanic patients from Colorado and New Mexico.
18. Northern analysis and RT-PCR detected aberrant splicing and mutations of TEG101 in human breast cancer cell lines.
19. We report on molecular cloning of a novel human cDNA by its interaction with the splice factor SRp30c in a yeast two-hybrid screen.
20. All exon-intron boundaries agree with GT-AG rule.
21. Using RT-PCR analysis, we show that human 20alpha-HSD, and PGFS mRNAs express ubiquitously, while DD4 mRNA is restricted to the liver.
22. Regions of strong divergence between chicken fast C-protein and human slow C-protein may represent differences in C-protein isoforms.
23. Identification of I-plastin, a human fimbrin isoform that is expressed in intestine and kidney.
