# Supplementary material for: Extraction of Transcript Diversity from Scientific Literature
Source: PLoS Comput Biol. 2005 Jun 24;1(1):e10. doi: 10.1371/journal.pcbi.0010010 (PMC1183516; doi:10.1371/journal.pcbi.0010010)
Supplement: Protocol S1 — (112 KB PDF). [file pcbi.0010010.sd001.pdf]

## Supplementary Information

### Description of the training set

The mechanisms for generating transcript diversity have been studied experimentally, using various biochemical methods including variants of PCR, S1 nuclease assays and blot hybridizations. The conclusion about the mechanism(s) involved, can be reached after nucleotide sequencing and computational analysis. Hence, sentences describing events that generate TD (Supp figure 3) may contain event mechanisms, results of the experimental methods or statements describing observations or presumptions.

The information generally available from these sentences includes gene names, experimental methods, tissue and species specificity, alternative exon function and other biologically interesting properties. The amount of information retrievable from different sentences varies much: from most of this information to a really partial one (categories 1-3; Supp. figure 3). For lack of space, absence of conclusive experimental evidence, or stylistic reasons, the event mechanism may not be mentioned in the abstract text (e.g., [1,2,3]). In these cases, the event may be missing but the presence of other word chunks may give enough bases to consider them positive sentences (category 3). Such events, which we aim to catch automatically, can be verified by manual/automated curation of article full-text, or computational analysis. For example, we detected sentences for DCLK gene, from the articles published in 2004 [4] and 1999 [5]. The article published by Engels *et al*., describes alternative splicing as the event that was perhaps not fully described by Sossey-Alaoui and co-workers. By using the event description our classifier detects the event.

### Part of speech tagging

The task of POS-tagging is to assign part of speech tags (e.g., verb or noun) to words reflecting their syntactic category.

**Inductive Learning**

In the process of inductive learning, positive and negative learning examples are provided to a learning method. The learning performance is then assessed on the set of examples the learner haven't seen before. The process is repeated till the classifier achieves satisfactory performance.

**Predicate argument structures**

A verb which indicates a particular type of event conveyed by a sentence can exist in its verbal form, its participial modifier format or its nominal form. For example, the normal form of a verb used to describe the event "finding presence of something" would be *detect*, its participial modifier format would be *detecting* or *detected*, and its nominal format would be *detection*. Sentence constituents holding meaningful roles to complete the meaning of an event indicated by the verb are called arguments. (also see below)

**Merging multiple syntactic patterns to semantic patterns**

For example, in the sentence, 'Northern blot analysis detected the presence of a 2.4kb transcript and a 3.2 kb transcript in brain, liver and pancreas', the phrases 'Northern blot analysis' and 'brain, liver and pancreas' would serve the role of arguments to the verb *detect* with semantic labels of *experimental methods* and *tissues*, respectively. It is clear that variation of the sentence as 'Detection of 2.4 kb and 3.2 kb transcripts present in brain, liver and pancreas by northern blot analysis' would not change the semantic role assigned to constituent 'northern analysis' and 'brain, liver and pancreas'. At the same time in sentence, 'Using RT-PCR and nucleotide sequencing, alternative splicing was confirmed in liver, brain and testis', phrases 'RT-PCR and nucleotide sequencing' and 'liver, brain and testis' would serve roles of *experimental methods* and *tissues*, respectively.

**Rules for extracting semantic patterns**

For example, a rule to find out the role of the variable region in alternatively spliced transcripts in terms of structure or function could be summarized as

follows: "*Take Noun phrase chunks right to different forms of verbs 'lack' (Figure 2; sentence 4) and 'differ'. Terminate when any of the end condition is encountered".* The end condition includes encounter of end of line, break in the sentence, different forms of 'be', words like 'through', 'due to' and 'because'. The rule for extracting experimental methods can be described as follows: "*Take chunks left to the different verbs 'show' and 'detect' (Figure 2; sentence 4, 6, 8, and 9) containing certain keys words (e.g., PCR or blot). Take the chunks to the right if passive form of verbs is used*".

Apart from the phrases extracted using predicate argument structure analysis, event mechanisms were extracted based on bi-gram and tri-gram lists. Tissue specificity was identified by tagging the word 'specific*' that may follow the tagged tissue name or part of the word describing the tissue (e.g. brain-specific). Similarly, 'number of isoforms' was extracted by the fact that such numbers always preceded the tagged event mechanisms. Tissues were tagged using a dictionary compiled from Swissprot and Refseq. Gene names were tagged using an entity tagger [6].

**Example entry from the database**

Information extracted from the Medline abstracts and different sequence databases is incomplete. Such incompletion resulted in variability in contents for our database entries. For example, information about AS in the human *neuropsin* has been well annotated in Swissprot and RefSeq (Supp. figure 1). Text extraction data in this case added information about tissue, experimental methods, and species-specificity observed in these alternative splicing events.

**Supplementary figure legends**

**Supplementary figure 1**: **An example database entry**

Entries in our database have three distinct parts. First part includes the pubmed identifier and title of the abstract. Second part contains mappings from sequence

databases like Swissprot, Refseq, GenBank and Ensembl. The third part includes knowledge derived from text with extraction rules.

**Supplementary figure 2: Distribution of results**

Figure 2a: The pie chart in the middle shows the number of abstracts that could be mapped to sequence databases using literature entries and synonymous list and those that couldn't (clockwise). The bar graph with categories 1-4 shows number of abstracts in which mechanism could be assigned to genes extracted from those abstracts. We have used MeSH terms and species information to identify gene studied in the abstract (bar graph with categories a, and b).

Figure 2b: We mapped all Swissprot, RefSeq and GenBank sequences to Ensembl genes for human, mouse and rat genomes. Using literature entries present in these databases we mapped our results to Ensembl genes. We could add 674, 637, and 359 annotations for AS for human, mouse and rat genomes, respectively.

**Supplementary figure 3: Description of training set**

Example sentences from our training set, describing generation of transcript diversity (figure3a) and negative sentences (figure3b) from MEDLINE. Alternative transcripts are generated by many mechanisms or combinations of them. Hence, the SVM classifier has to learn multiple patterns apart from their syntactic variants. The sentences are classified in to various categories and semantic patterns are marked from 1-8. Please see table1 for the pattern labels.

**References**

1. Rajavashisth TB, Eng R, Shadduck RK, Waheed A, Ben-Avram CM, et al. (1987) Cloning and tissue-specific expression of mouse macrophage colony-stimulating factor mRNA. Proc Natl Acad Sci U S A 84: 1157-1161.
2. Russell DL, Ochsner SA, Hsieh M, Mulders S, Richards JS (2003) Hormone-regulated expression and localization of versican in the rodent ovary. Endocrinology 144: 1020-1031.
3. Lonnerberg P, Ibanez CF (1999) Novel, testis-specific mRNA transcripts encoding N-terminally truncated choline acetyltransferase. Mol Reprod Dev 53: 274-281.
4. Engels BM, Schouten TG, van Dullemen J, Gosens I, Vreugdenhil E (2004) Functional differences between two DCLK splice variants. Brain Res Mol Brain Res 120: 103-114.
5. Sossey-Alaoui K, Srivastava AK (1999) DCAMKL1, a brain-specific transmembrane protein on 13q12.3 that is similar to doublecortin (DCX). Genomics 56: 121-126.
6. Mika S, Rost B (2004) Protein names precisely peeled off free text. Bioinformatics 20 Suppl 1: I241-I247.
